# Epigenetics, DNA damage, and aging

**DOI:** 10.1172/JCI158446

**Published:** 2022-08-15

**Authors:** Carolina Soto-Palma, Laura J. Niedernhofer, Christopher D. Faulk, Xiao Dong

**Affiliations:** 1 Institute on the Biology of Aging and Metabolism,; 2 Department of Biochemistry, Molecular Biology, and Biophysics,; 3 Department of Animal Science, and; 4 Department of Genetics, Cell Biology and Development, University of Minnesota, Minneapolis, Minnesota, USA.

## Abstract

Over the course of a human lifespan, genome integrity erodes, leading to an increased abundance of several types of chromatin changes. The abundance of DNA lesions (chemical perturbations to nucleotides) increases with age, as does the number of genomic mutations and transcriptional disruptions caused by replication or transcription of those lesions, respectively. At the epigenetic level, precise DNA methylation patterns degrade, likely causing increasingly stochastic variations in gene expression. Similarly, the tight regulation of histone modifications begins to unravel. The genomic instability caused by these mechanisms allows transposon element reactivation and remobilization, further mutations, gene dysregulation, and cytoplasmic chromatin fragments. This cumulative genomic instability promotes cell signaling events that drive cell fate decisions and extracellular communications known to disrupt tissue homeostasis and regeneration. In this Review, we focus on age-related epigenetic changes and their interactions with age-related genomic changes that instigate these events.

## Introduction

**Figure 1:**
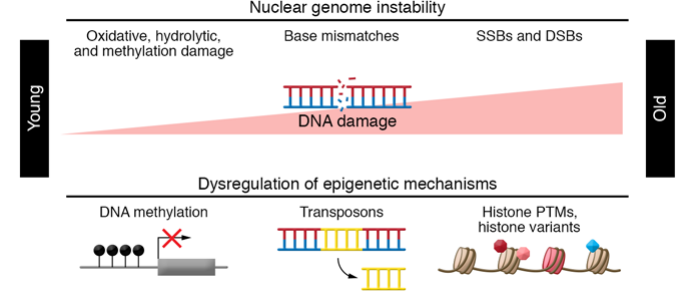
Epigenetics, DNA damage, and aging. Aging is determined, in part, by interrelated mechanisms that affect nuclear genome integrity: macromolecular damage to DNA and epigenetic alterations. DSBs, double-strand breaks; PTMs, posttranslational modifications; SSBs, single-strand breaks.

The biology of aging is very complex, and the heterogeneity of aging is abundantly clear. Over a decade ago, nine hallmarks of aging were identified at the cellular and molecular level (1). The universality of the hallmarks of aging across species suggests their causal role in driving aging. However, establishing cause and consequence has proved challenging. Notably, more than one of the hallmarks reflect alterations to the nuclear genome, the integrity of which is vital to cell function (2). Here, we focus on the relationship of two hallmarks of aging affecting the nuclear genome: macromolecular damage and epigenetic alterations (Figure 1).

In eukaryotes, epigenetic modifications are critical because of their effects on gene transcriptional regulation. During development, different cell types establish and maintain specific epigenetic landscapes that dictate their cell fate. With aging, pronounced epigenetic alterations occur (3–6), including changes to DNA methylation and histone modifications, two key regulators of gene expression. Concurrent with these changes, spontaneous DNA lesions occur every single day within each of the 10^13^ cells that constitute a human body (7). These lesions stall DNA and RNA polymerases, provoking a DNA damage response (DDR) that halts the cell cycle, enabling DNA repair (8). Excessive or chronic DDR triggers irrevocable cell fate decisions, e.g., apoptosis and senescence. These two hallmarks of aging are intimately intertwined: DNA repair alters the epigenome and the epigenome impacts DNA repair efficiency. Furthermore, epigenetic marks to DNA can promote DNA damage (see the following section). Genotoxic stress (DNA damage) is accepted as playing a causal role in cancer and in aging (2, 9–13). Epigenome instability is established to play a causal role in cancer, but the mechanism by which epigenetic changes might play a causal role in aging are not well defined. Given the plethora of epigenetic clocks that correlate with chronological and even biological age, the causal relationship likely exists. Herein, we examine the current state of evidence that epigenetic alterations contribute to driving aging biology. In addition, because epigenetic changes impact genome stability, we explore the relationship between epigenetic marks and DNA damage.

## DNA methylation

“DNA methylation” typically refers to the addition of a methyl group to the C-5 position of a cytosine, although other modifications to the nucleic acids, e.g., DNA adenine methylation, have been shown to have significant biological function (14). DNA methylation can be established, recognized, and removed by enzymes classified according to their roles in this process — e.g., DNA methyltransferases (DNMTs) as writers that establish methylation, methyl-CpG-binding domain proteins (MDB proteins) as readers that recognize methylated nucleotides, and ten–eleven translocation (TET) enzymes as erasers that remove DNA methylation (15). In mammalian cells, most DNA methylation occurs in CpG dinucleotides and is profoundly remodeled during cell differentiation (16, 17). Non-CpG methylations are found in oocytes and embryonic stem cells (18, 19), but are rare in mature and differentiated cell types. In a human genome, there are approximately 28 million CpG dinucleotides, unevenly distributed throughout the genome. Regions with a high frequency of CpGs are called CpG islands (CGIs) (20), which are hundreds to thousands of base pairs in length. CGIs are often located in or near gene promoter regions. Methylation of CpGs in these CGIs or promoters negatively correlates with transcription activity of the local genes. In contrast, gene body methylation shows a bell-shaped correlation with gene expression: modestly expressed genes have the highest methylation level in their gene body, while both weakly and highly expressed genes show low levels of gene body methylation (21).

### Epigenetic clocks

Based on cross-sectional comparison of subjects of different ages, DNA methylation is altered with aging. Early studies suggested a loss of global methylation during aging. However, more recent studies indicate that either hypermethylation or hypomethylation may occur in different areas of the genome and that approximately 2% of the CpG sites in the genome change with age in different tissues of humans and mice (22). Using methylation status of only a small percentage of the age-related CpG sites, accurate prediction models for chronological age have been built, known as epigenetic clocks. The most cited clock for humans is composed of 353 CpG sites and was developed based on 8000 samples of 51 healthy tissues and cell types (23). These epigenetic clocks have been found to be relevant to biological age as well. For example, progeroid syndrome patients show an average of 6.4 years of accelerated clock age (24). In a cohort of 1863 older adults, subjects with accelerated epigenetic clock age exhibited a higher risk of cancer, cardiovascular disease, and all-cause mortality (25). Notably, clock age can also be decelerated by calorie restriction in mice (26) and rejuvenated to a younger age in fibroblasts after reprogramming to a pluripotent state (27). In naked mole rats, an emerging popular model for aging studies because of their exceptional maximum lifespan (37 years), breeding queens age more slowly than nonbreeders according to their epigenetic clock (28). Ectopic expression of three of the four Yamanaka factors (*OCT4*, *SOX2*, *cMYC*, and *KLF4*), which regulate pluripotency in embryonic stem cells, in old mice reverses vision loss (associated with biological age) and restores youthful DNA methylation patterns (29). While epigenetic clocks are compelling and useful, their mechanistic link to aging remains elusive (30).

**Figure 2:**
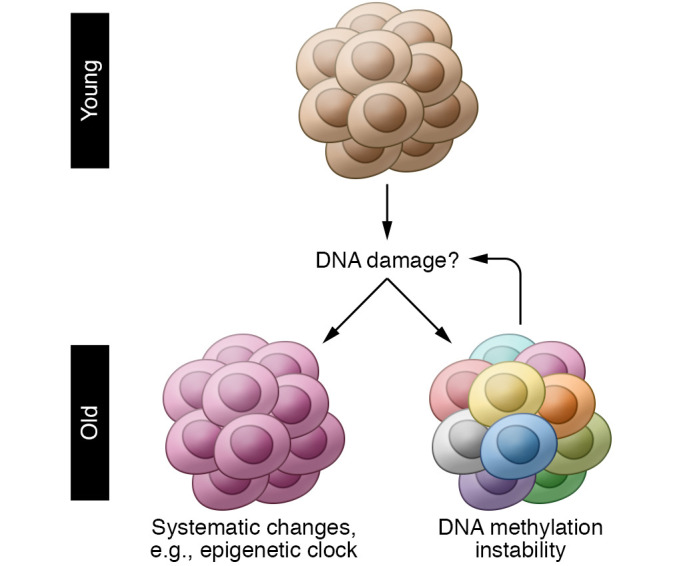
DNA damage and age-related DNA methylation alterations. DNA damage has been recognized as a significant causal factor in age-related functional decline. Recent evidence has suggested its impact on age-related systematic and stochastic changes in DNA methylation (e.g., advancing the epigenetic clock and epigenetic drift).

If DNA damage and age-related epigenetic changes are linked, a prediction is that increased DNA damage would accelerate the epigenetic clock (Figure 2). DNA repair deficiency can cause premature aging in humans and mice, e.g., XFE progeroid syndrome, caused by mutations in *XPF*, which encodes the catalytic domain of the DNA repair endonuclease XPF-ERCC1 (31). Indeed, genome instability is linked to most, if not all, human progeroid syndromes (32). DNA repair deficiency leads to the accumulation of endogenous DNA lesions (33), and excessive DNA damage can cause senescence at the cellular level (34). However, there is a limited number of studies on the effect of DNA damage on the epigenetic clock, and they report conflicting findings. Human endothelial cells exposed to ionizing radiation become senescent, but their epigenetic clock age does not increase (35). In contrast, induction of DNA double-strand breaks (DSBs) in mice does advance the epigenetic clock (36).

### DNA methylation instability

DNA damage is largely viewed as stochastic, occurring randomly throughout the genome. In contrast, the reproducibility of epigenetic clocks intimates that epigenetic changes are not random. This suggests that either the two pillars of aging have not been adequately analyzed, particularly at the single-cell and same-cell levels, or that the two pillars of aging are not entirely codependent. The latter appears unlikely, based on the following: Homologous recombination (HR) repair of DSBs impacts local DNA methylation (and histone H3 methylation). In two identical cells, induction of DSBs within the same genetic locus leads to differences in DNA methylation status between the cells (37) and, as a consequence, transcriptional heterogeneity between them. This demonstrates that DNA damage promotes epigenome instability (Figure 2). It is important to note that endogenous DNA damage, caused by the chemical instability of DNA under physiological conditions, leading to its hydrolysis, oxidation, and methylation, results in tens of thousands of lesions per day, per cell (7), affording countless opportunities for activation of the DDR, mutagenesis, epigenetic alterations, and cell-fate decisions across the lifetime of a cell and an organism.

In addition to DNA damage causing epigenetic changes, the converse is also true. 5-Methylcytosine can be spontaneously oxidized or enzymatically modified by the TET enzymes to form 5-hydroxymethylcytosine and 5-formylcytosine. Both oxidized bases are substrates for base excision repair (BER) (38, 39), yet they also act as epigenetic marks, regulating gene expression with a role in human neurodevelopmental and neurodegenerative disorders (40). BER of these oxidized bases functions as a demethylase (eraser), a regulatory mechanism of DNA methylation and gene expression. 5-Hydroxymethylcytosine appears to be localized at sites of DNA damage (41), which may help identify sites requiring repair (41), consistent with the finding that abundance of this modified base is inversely correlated with mutation frequency (41). In contrast, 5-formylcytosine may be mutagenic (39). Collectively, these nascent studies illustrate the complex relationship between genome and epigenome stability.

Instability in DNA methylation has been technologically difficult to define. As a surrogate to single-cell analysis, monozygotic twins have been used to study DNA methylation and histone acetylation (42, 43). At a young age, monozygotic twins show remarkable similarity in epigenetic marks, while older twins show significant epigenetic difference or “drift.” Another approach to estimating cellular epigenetic heterogeneity is based on informatics analysis of bulk tissue bisulfite sequencing reads. The number of different patterns of methylation of multiple CpGs in multiple bulk analyses can be used to estimate DNA methylation heterogeneity (44, 45). By this approach, increased DNA methylation heterogeneity is found in several cancers, including acute myeloid leukemia, chronic lymphatic leukemia, Ewing sarcoma, and glioblastoma, and correlates with clinical outcomes (45–49). But the extent of DNA methylation heterogeneity that occurs with aging remains to be defined.

Of course, single-cell analysis is the ideal approach to define cell-to-cell epigenetic variation. Using single-cell bisulfite sequencing, we observed significant DNA methylation heterogeneity in hepatocytes of mice, and the heterogeneity appears to increase with animal age (50). In mouse muscle stem cells, an age-related increase in methylation heterogeneity is found in gene promoter regions across the genome (51) and is likely to contribute to the elevated transcriptional noise that occurs with aging (51–54). However, like the epigenetic clock, the link between age-related DNA methylation heterogeneity and DNA damage remains untested. With the rapid improvement in the cost of next-generation sequencing, we believe that this question will be resolved with the application of single-cell multi-omics approaches.

## Transposons

**Figure 3:**
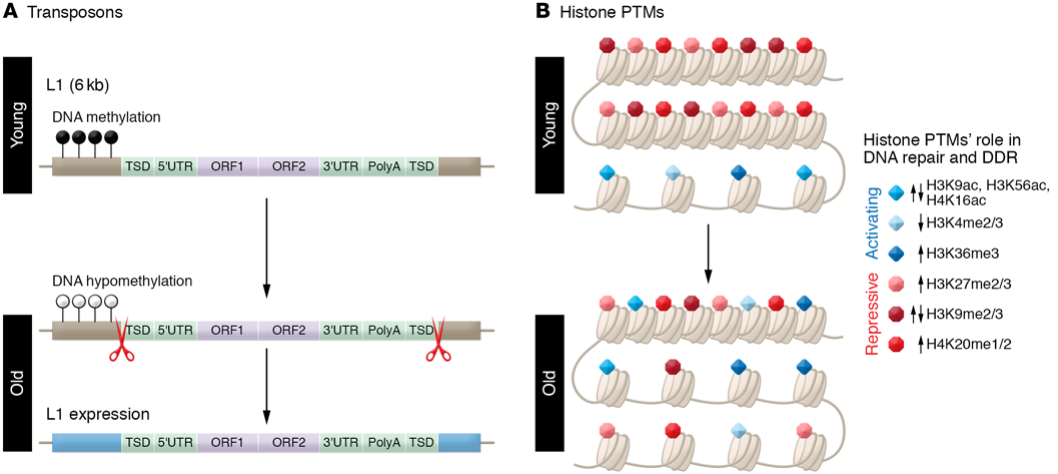
Age-related changes in transposons and histone marks. With aging and senescence, L1s become demethylated and thereby derepressed. Activation of the L1s leads to additional genome instability as the genetic elements reinsert into the genome. Additionally, they promote cytosolic DNA fragments, which activate cGAS-STING, inflammation, and senescence. At the histone level, histone PTMs that regulate chromatin compaction, gene expression, and the ability to repair DNA damage change with aging. Generally speaking, with aging, repressive marks are lost and activating histone PTMs increased. This can promote prolonged activation of the DNA damage response (DDR), transcriptional dysregulation, senescence, and inflammation.

Transposons play a role in many aspects of genome regulation due to their sheer prevalence, accounting for about 50% of the genome in mammals. DNA methylation works in concert with histone modifications as a mechanism for heterochromatinization of transposons to repress transposon expression and activity, which is critical for the maintenance of genome stability. This heterochromatinization becomes progressively disorganized with age. The tendency toward a global loss of DNA methylation with aging results from the fact that most DNA methylation occurs in intergenic regions and specifically in transposons owing to their need to be silenced (Figure 3). CpGs found within transposons tend to lose methylation with organism age, in contrast to promoter CpGs that gain methylation with age, resulting in an overall loss of global DNA methylation. This “regression to the mean” was seen even within transposon families where weakly methylated smaller fragments gained methylation with age in mice, while larger (>5 kb), more highly methylated fragments lost methylation (55). Here, we discuss what is known about epigenetic regulation of the classes of various transposons and its impact on aging, DNA damage, DDR, and aging (Table 1).

### LINE-1 elements

Age-related changes in DNA methylation at transposons have a variety of impacts on gene expression. LINE-1 elements (L1s) are shared across vertebrates and make up 21% of the human genome, which contains about 100,000 truncated copies, about 4000 full-length copies, and about 100 “hot L1s,” capable of mobilization. L1s are generally enriched in intergenic regions and depleted in promoter regions. During aging and cellular senescence, L1s become demethylated and derepressed (Figure 3) (56). In late senescence, L1 transcription activates a type I interferon (IFN-I) response that contributes to the maintenance of the senescence-associated secretory phenotype (SASP) (57). L1 demethylation patterns seen in cancer are recapitulated during normal aging, suggesting a mechanistic link between transposon methylation and increasing cancer risk with age (58). L1 methylation is inversely associated with inflammation markers such as vascular cell adhesion molecule-1 (VCAM-1) (59) and is associated with incidence of prostate cancer (60), a highly age-related disease. While the global pattern of L1 methylation loss is consistent, individuals diverge in unique ways, as shown by age-dependent divergence of L1 methylation in a study of monozygotic twins (61). L1 hypomethylation is linked to the presence of centromere-positive micronuclei, which are indicators of age-related genomic instability (62).

### Alu sequences

The human genome contains 1.5 million Alu elements, which, in contrast to L1s, are shorter and biased toward euchromatic regions, along with having a much higher CpG density. Alus are primate specific and continue to actively mobilize within humans. Alu methylation is associated with normal and pathogenic aging phenotypes. Epidemiological studies indicate that Alu methylation in blood cells is directly correlated to bone mass density, independent of age (63). Offspring (>65 years old) of healthy nonagenarians maintain higher levels of Alu methylation than age-matched controls, with diets high in fruits and whole grains showing an interaction effect (64). In human cohorts, Alu methylation is associated with all-cancer mortality in an age-dependent manner (60). At a cellular level, Alu methylation is protective against DNA damage. This was tested with the use of siRNA of DMTs to increase methylation, which corresponded with improved resistance to DNA damaging agents and increased proliferation in vitro (65).

### Relationship to DNA damage and repair

DSB repair rates within various classes of repeats are associated with the chromatin conformation of those repeats. Euchromatic Alu sequences are replicated in early S phase, while L1s have a more elongated replication window throughout S phase, and satellite IIIs, which are highly heterochromatinized around centromeres, are replicated exclusively in mid– to late S phase (66). Accordingly, DSBs occurring in Alu sequences follow global repair kinetics while DSBs in L1s are repaired more slowly, likely as a result of histone-driven euchromatinization. In Fanconi anemia (FA), a genome instability disorder caused by defective repair of DNA interstrand cross-links, derepression of multiple classes of transposons is observed, and FA protein complex components are localized to L1 transposons in mouse embryos (67).

Studies on age-related changes to DNA methylation in other transposon families are sparse. One study reported age-related loss of DNA methylation in Alu and human endogenous retrovirus K, both evolutionarily young families, but did not replicate L1 hypomethylation seen in other studies, perhaps because of the insensitivity of the assay used (68). Mouse B1 elements are analogous to human Alu elements, having the same 7SL RNA progenitor. Intracisternal A-particle (IAP) elements are the most active class within mice. While both B1 and IAP elements follow typical patterns of age-related DNA hypomethylation, a longitudinal study showed that this rate was not disrupted by low-dose radiation even 19 months after irradiation (69). Overall, more knowledge is needed to understand aging DNA methylation dynamics of all classes of transposons, considering that even some of the smaller overlooked classes nevertheless comprise as much or a greater percentage of the genome than all coding regions combined. Likewise, their impact on DNA repair and the DDR is poorly understood since transposons are difficult to study owing to challenges in mapping them within the genome. Long-read sequencing and newer methods of DNA modification detection will be vital to advancing the field.

## Histone posttranslational modifications

In addition to DNA methylation, epigenetic modifications of histones play an important role in DNA repair, the DDR, and aging. These modifications, such as phosphorylation, acetylation, and methylation, remodel the chromatin structure to enable transcription as well as DNA repair (70, 71). For instance, H2AX with phosphorylated serine-139 residue, i.e., γH2AX, at a DSB locus recruits DSB repair enzymes to the damage site, promoting repair as well as transduction of DDR signals (72). Histone modifications become significantly altered with age (3, 4). Here, we discuss each type of histone modification and briefly describe the roles of chromatin remodeling complexes and histone variants in the DDR and aging in mammalian systems (Table 2).

### Histone acetylation

Histone acetylation can occur on lysine residues of H2A, H2B, H3, and H4. Acetylation disrupts the interaction between the histones and DNA, loosening chromatin structure, and activating transcription of local genes. The processes of histone acetylation and deacetylation are catalyzed by enzymes, known as the histone acetyltransferases (HATs) and histone deacetylases (HDACs), respectively. Histone acetylation also plays an important role in DDR (73, 74). In response to DNA damage, depending on the specific type and amount of DNA damage, hyperacetylation or hypoacetylation of lysine residues of histones can occur locally, allowing repair proteins to gain access to the DNA. After DNA repair, the histones are restored to their original PTM state (73, 74). The HAT TIP60 acetylates H4K16, facilitating HR repair (75). HDAC1 and HDAC2 play important roles in DSB repair by deacetylating H3K56ac and H4K16ac (76, 77). H4K16ac blocks 53BP1 DNA binding, which promotes HR-mediated repair of DSBs (75). Deacetylation of H4K16ac by HDAC1 and HDAC2 promotes nonhomologous end joining (NHEJ) of DSBs (76). In accordance, HDAC inhibitors block NHEJ of telomeric ends (78). Therefore, acetylation/deacetylation dynamics dictate DSB repair pathway choice.

SIRT6 is an NAD^+^-dependent H3K9 HDAC, which, among its other enzymatic activities, is linked to longevity in mammals (79, 80). Genetic depletion of SIRT6 leads to telomere dysfunction and premature cellular senescence (81, 82). SIRT6 interacts with the NF-κB RELA subunit and deacetylates H3K9 at NF-κB target gene promoters, dampening NF-κB signaling and attenuating cellular SASP (83). SIRT6 also deacetylates H3K56 found at DSB sites, along with HDAC1 and HDAC2, to promote chromatin restoration and genome stability (84). Moreover, species-specific variants of SIRT6 are responsible for more efficient DSB repair in long-lived species (85, 86).

A comparison of human brain samples among young and old subjects and Alzheimer’s disease (AD) patients revealed important differences in H4K16ac (87). In old brain samples, H4K16ac is increased; however, in AD patients, there is a global decrease of H4K16ac. Specifically, there is a loss of H4K16ac in regulatory regions of the genes specifically expressed in neurons, and a conserved pattern of transcriptional changes among different AD patients. These transcriptional changes are related to myeloid differentiation, cell death, and WNT and RAS signaling, suggesting the existence of an AD-specific transcriptional program (87). Expression of the transcriptional corepressor REST increases with age in neuronal populations of the human brain but decreases in AD, which suggests a protective role for this complex. REST deacetylates H3K9ac. Thus H3K9ac levels are globally downregulated in normal aging neurons, but increased in AD patients, in whom it has been shown that there is a derepression of genes that promote AD pathology (88). Collectively, these studies clearly demonstrate that histone acetylation/deacetylation plays a key role in preserving genome stability and epigenetic homeostasis in aging and age-related diseases.

### Histone methylation

Histone lysine methylation regulates transcription through effector molecules known as readers, which specifically recognize methylated sites. Generally, methylation at H3K4 and H3K36 marks (denoted as H3K4me and H3K36me) is positively correlated with active transcription, whereas H3K9me, H3K27me, and H4K20me are associated with silenced chromatin states (89). Mono-, di-, and trimethylation can occur via activity of histone lysine methyltransferases (KMTs), referred to as writers, and histone lysine demethylases (KDMs), which are known as erasers (89). Near DSB sites, in general, there is a depletion of the activating marks H3K4me2/3, and a gain of the repressive marks H3K9me2/3, H3K27me3, and H4K20me3 (90). However, the presence of H3K36me3, a mark found in the gene bodies of actively transcribed genes, appears to be necessary for DNA repair as well (90). With aging, there is generally a loss of repressive marks and a gain of activating marks, perhaps contributing to decreased DNA repair and increased transcriptional dysregulation (3–6).

#### H3K4.

H3K4me1, H3K4me2, and H3K4me3 are markers for enhancers, gene 5′ ends, and active promoters, respectively. Overall, at sites of DNA damage, there is a reduction of H3K4me2/3 mediated by KDMs. The bromodomain protein ZMYND8 is an important DDR factor that recruits the NuRD (nucleosome remodeling and histone deacetylation) complex to damaged DNA sites. Demethylation of H3K4me3 by KDM5A is required for ZMYND8-NuRD binding to damaged DNA sites, where the latter represses transcription and promotes DNA repair (91). KDM5B is required for efficient DSB repair through recruitment of Ku70 and BRCA1, which are essential components of NHEJ and HR, respectively (92). KDM1A, also known as LSD1, interacts with the E3 ubiquitin ligase RNF168 and is recruited to DSBs, where it promotes H3K4me2 demethylation and ubiquitination of H2A/H2AX, thereby facilitating 53BP1 recruitment (93). A comparison of young and old murine hematopoietic stem cells (HSCs) showed that aged HSCs exhibit broader H3K4me3 peaks across HSC identity and self-renewal genes (the latter allow stem cells to divide by maintaining their stemness) (94). Therefore, these changes in H3K4me3 accompanied by changes in DNA methylation reinforce a relationship between diminished differentiation and HSC age (94). In senescent cells, there are genome-wide alterations in H3K4me3 domains referred as “mesas” (95). H3K4me3 mesas form part of the chromatin reorganization observed in cells of Hutchinson-Gilford progeria syndrome (95), a disease of accelerated aging comprehensively reviewed in ref. 10 and ref. 96.

#### H3K36.

H3K36me3 is a mark enriched on the gene bodies of actively transcribed genes. H3K36me3 modulates transcriptional fidelity by regulating RNA polymerase II elongation and preventing cryptic transcription (97, 98). H3K36me3 mark is also implicated in mRNA splicing, as exons are preferentially marked with H3K36me3 compared with intronic sequences (99). In DNA mismatch repair (MMR), H3K36me3 helps to recruit the mismatch recognition protein hMutSα (hMSH2-hMSH6) to sites of nucleotide mismatches through direct interactions with the hMSH6 PWWP domain (100, 101). Depletion of SETD2, the main H3K36me3 trimethyltransferase, impedes HR repair of DSBs in transcriptionally active chromatin (102–104). Overexpression of KDM4, an H3K36me3 demethylase, disrupts MMR (105). In addition, SIRT6 mono-ADP ribosylates the H3K36me2 demethylase KDM2A, leading to increased H3K36me2 levels and recruitment of NHEJ proteins to DSBs (86). Decreased H3K36me3 has been found in brain tissue of aged senescence-accelerated mouse prone 8 (SAMP8) mice compared with wild-type controls (106). Loss-of-function mutations in the H3K36 histone methyltransferase NSD1 accelerate the Horvath epigenetic clock (107).

#### H3K27.

H3K27me3 is associated with facultative heterochromatin and transcriptionally silenced chromatin regions. Polycomb group (PcG) proteins function as transcriptional repressors that associate into two distinct classes of multimeric complexes, termed Polycomb repressive complex 1 (PRC1) and PRC2. Polycomb group members are recruited by poly(ADP-ribose) polymerase (PARP) to DNA lesions along with the NuRD deacetylase complex (108). Depletion of EZH2, an H3K27me2/3 methyltransferase and a part of the PRC2 complex, decreases DSB repair efficiency and increases radiation sensitivity (109). During aging, EZH2 levels decline in islet β cells, which coincides with loss of H3K27me3 at the INK4a/ARF locus, and increased expression of *p16^INK4a^* and *p19^ARF^*, limiting β cell regeneration in aged mice (110). Moreover, in replicative senescence and oncogene-induced senescence, there are large-scale domains of H3K27me3-depleted “canyons” mainly at genes and enhancers, which correlate with upregulation of key senescence genes (95).

H3K27me controls the gene expression dynamics that occur during osteogenic, adipogenic, and chondrogenic differentiation of multipotent mesenchymal stem cells (MSCs) (111). Osteoporosis is an age-related degenerative bone disease mediated at least in part by impaired osteogenic differentiation of bone marrow–derived MSCs. EZH2 expression is significantly increased in MSCs from osteoporotic mice (112). Therefore, inhibiting the histone methyltransferase EZH2 derepresses WNT signaling and improves osteogenic differentiation (112). In addition, overexpression of the anti-aging protein Klotho in early postnatal myogenesis downregulates the H3K27 demethylase KDM6B/JMJD3, thereby increasing H3K27me levels and suppressing WNT signaling, which delays muscle differentiation and increases muscle stem cells (MuSCs) needed for mammalian skeletal muscle regeneration (113). In addition, increased HOX9 expression in MuSCs from aged mice, which results from a gain in activating marks, causes the induction of developmental pathways that further inhibit MuSC function in aged muscle (114). H327me3 along with other histone marks clearly plays an important role in genomic and epigenome stability. However, the beneficial or detrimental role of this epigenetic mark greatly depends on the context.

#### H3K9.

H3K9me2/3 marks are associated with gene repression and constitutive heterochromatin. Restoration of H3K9 methylation following DNA repair is an important part of the DDR. For instance, a complex containing KAP-1, HP1, and the H3K9 di- and trimethyltransferase SUV39H1/KMT1A is rapidly loaded onto the chromatin at DSBs, thereby allowing transient formation of repressive chromatin (115). Domains of H3K9me3 are critical for TIP60 acetyltransferase activation, which acetylates ataxia telangiectasia–mutated (ATM) kinase and histone H4. Cells lacking SUV39H1 show defective activation of TIP60 and ATM, decreased DSB repair, and increased radiosensitivity (115). Similarly, the macrohistone variant macroH2A1 and H3K9 di- and trimethyltransferase PRDM2/KMT8A promote an ATM-dependent chromatin recondensation at DSBs (116). Chromatin expansion occurs independently from ATM signaling and instead involves PARP-induced chromatin remodeling, which promotes the recruitment of the H3K9 di- and tri-demethylases KDM4B/JMJD2B and KDM4D/JMJD2D to sites of DNA damage, thereby allowing efficient DSB repair (117, 118).

Besides its role in DDR, H3K9 methylation plays a key role in aging and cellular senescence. SUV39H1 levels decrease in cells exposed to ionizing radiation (119), diminishing H3K9me2/3 levels, and leading to de-heterochromatinization of satellite regions and induction of senescence markers (119). These results were confirmed in a model of replicative senescence in human lung fibroblasts (120). SUV39H1 has also been found to decrease with aging in both human and mouse HSCs. Its decrease leads to a reduction in HSC capacity to generate B lymphocytes, which decline with aging (121). Re-expression of SUV39H1 improves the capacity of HSCs from elderly individuals to generate B cells (121). In oncogene-induced senescence, there is proteasomal degradation of the H3K9 mono- and dimethyltransferases G9a and GLP, mediated by the activation of APC/C^Cdh1^ ubiquitin ligase. G9a and GLP depletion causes a global decrease in H3K9 dimethylation at promoters of IL-6 and IL-8 and other SASP-related genes, thus inducing increased transcription of these factors (122). G9a and H3K9me1/2 levels have also been found to be downregulated in a rat model of age-related hearing loss (123). In addition, depletion of the H3K9 di- and trimethyltransferase SETDB1/KMT1E attenuates H3K9me2/3 levels and augments TLR4-mediated NF-κB recruitment to the proximal promoter region of IL-6, thereby inducing its transcription (124). Along the same lines, macrophage-specific SETDB1-knockout mice exhibit higher serum IL-6 in response to LPS challenge and are more susceptible to endotoxin shock than wild-type mice (124). Finally, the H3K9me3 demethylase KDM4 is upregulated in oncogenic, replicative, and genotoxicity-induced senescence (125). Notably, removal of KDM4, either genetically or using an inhibitor, rescued H3K9me3 levels and abrogated SASP expression (125). Collectively, these studies illustrate the importance of preserving H3K9 methylation levels to assure an efficient DDR and to prevent a proinflammatory SASP.

#### H4K20.

H4K20me3 is a mark of silenced heterochromatic regions (126). SET8/PR-Set7 is the sole H4K20 monomethyltransferase. It is transiently recruited to sites of DNA damage through its interaction with proliferating cell nuclear antigen (PCNA), thereby facilitating 53BP1 recruitment (127). Deletion of H4K20 di- and trimethyltransferases SUV4-20H1 and SUV4-20H2, in hematopoietic cells alters chromatin structure and increases chromosomal aberrations in B cells (128). Activated TGF-β signaling in response to oxidative stress suppresses SUV4-20H via microRNA-29 activity (129). SUV4-20H suppression reduces H4K20me3 levels, which compromises DDR and genome maintenance, contributing to cardiac aging in vivo (129).

### Histone variants

The H2A variants H2AX, H2AZ, and macroH2A, along with H3 variant H3.3 promote genome stability by participating in DDR pathways, as recently reviewed in ref. 130. Defects in H2A variant function in humans have been shown to lead to diseases including cancer as well as aging. Phosphorylation of H2AX on serine-139, known as γH2AX, in response to DSBs, is a well-known marker of DNA damage recognition, repair, and resolution or initiation of cellular fates in response to DNA damage. H2AX-null mice are smaller than their WT littermates and are more sensitive to Iionizing radiation (131). MacroH2A1 is required by KDM5A, and H3K4 demethylase, to recognize DNA lesion sites (132). MacroH2A accumulates in human fibroblasts undergoing replicative senescence and in tissues of aged mice and primates (133). Conversely, in cellular senescence macroH2A1 is removed from SASP genes, contributing to the activation of proinflammatory cytokine production (134). Removal of macroH2A1 from SASP genes is mediated by activation of ATM in response to endoplasmic reticulum stress (134). H3.3 is enriched in transcriptionally active chromatin regions and is increased in various tissues of aged mice (135). H2AZ increases during aging, and it has been found to be reduced during active learning in aged mice (136).

### Chromatin remodelers

The reorganization of chromatin architecture is mediated by ATP-dependent chromatin remodeling complexes such as the switch/sucrose non-fermentable (SWI/SNF) complex. The SWI/SNF complex is recruited to γH2AX foci and required for efficient recruitment of NHEJ proteins (137). In addition, the two catalytic ATPase subunits of the SWI/SNF complex, BRM and BRG1, are implicated in the maintenance of telomere structure and function (138).

### Histone modification in progeria models

Hutchinson-Gilford progeria syndrome (HGPS) is a premature aging disorder caused by a point mutation in the *LMNA* gene, one of the major architectural elements of the mammalian cell nucleus, leading to the production of a mutant form of lamin A called progerin. Progerin sequesters replication and repair factors, leading to DNA replication defects, which are a prominent source of DSBs (139). In addition, DSB repair is impaired by a delay in recruitment of DNA repair proteins (140). Other progeroid syndromes, like Werner syndrome, Bloom syndrome, and Cockayne syndrome, are more directly linked to genome instability through mutation of DNA repair genes, including Werner syndrome helicase (*WRN*).

Interestingly, cells from patients with HGPS resemble chromatin defects characteristic of physiological aging, such as loss of heterochromatin, loss of repressive marks, downregulation of the heterochromatin protein HP1, and increased transcription of pericentromeric satellite III repeats (141). Similarly, in WRN-deficient MSCs there is a reduction of the heterochromatin-associated inner nuclear membrane (INM) proteins, as well as downregulation of the constitutive heterochromatin mark H3K9me3 (142). Moreover, loss of several components of the NuRD complex and a reduction in its catalytic HDAC1 occur in both premature and normal aging (141). Taken together, these results suggest that perturbed DNA repair is a cause of further loss of epigenetic homeostasis. Conversely, Pegoraro and colleagues have proposed that epigenetic changes may lead to genomic instability based on the observation that silencing of individual NuRD subunits recapitulates chromatin defects associated with aging that precede DNA damage accumulation (141).

## Conclusion

In summary, the comprehensive interactions between DNA damage and the aging epigenome occur at multiple epigenetic layers and bidirectionally. Remodeling of local epigenetic modifications is an essential step in the comprehensive network of DNA damage repair, while insufficiency in restoring original epigenetic marks to the damaged sites after repair is likely a significant source of the pronounced epigenetic alterations observed during aging. To understand the complete landscape of the intertwined relationship between these two major hallmarks of aging will require substantial additional research effort. Key areas of uncertainty that require further investigation include resolving the relationship between DNA damage and epigenetic changes at the single-cell level, and determining the predominant mechanisms by which DNA damage and epigenetic changes promote aging. These mechanisms may differ by cell type. Despite the lack of precise mechanism, there are approaches available to therapeutically modify epigenetic changes. This is exciting, as it may prove more tractable than trying to prevent endogenous DNA damage. Like other therapeutic approaches targeting fundamental aging processes, drugs targeting epigenetic changes are being developed for age-related diseases rather than aging itself (see below). This is for practical and regulatory reasons. Nevertheless, establishing that these approaches are safe and mechanistically efficacious in humans is a critical step toward their implementation to disrupt the biology of aging.

## DNA methyltransferase inhibitors

DNA methyltransferase inhibitors (DNMTis) restore the function of genes aberrantly silenced by DNMTs, as reviewed in ref. 143. DNMTis are classified into two categories, nucleoside and non-nucleoside inhibitors. Nucleoside inhibitors include 5-azacytidine (5-Aza) and 5-aza-2′-deoxycytidine (decitabine). Curcumin and procaine are non-nucleoside inhibitors. 5-Aza and decitabine are routinely used to treat myelodysplastic syndrome, a group of rare blood cancers, and are well tolerated. Clinical trials (phase II, III, and IV) yielded a dramatic antitumor effect of 5-Aza on recurrent gliomas harboring isocitrate dehydrogenase (*IDH1* or *IDH2*) mutations. Non-nucleoside inhibitors of DNMT, such as RG108, are being developed for their antitumor properties, for instance in promoting apoptosis of endometrial and esophageal cancer cells (144, 145).

### Histone deacetylase inhibitors

Valproic acid (VPA) is an antiseizure medication, also used to treat delirium, which is well tolerated and efficacious. VPA has HDAC inhibitor (HDACi) activity as well as other properties impacting neurochemical and cellular abnormalities. HDACis have shown encouraging results in preclinical models of cancer, but clinical trials yielded only limited success. Combinatorial regimes with other cancer chemotherapeutics revealed synergistic effects in both preclinical and clinical studies, reviewed in ref. 146, suggesting that HDACis may reduce toxicity and resistance to standard cancer therapy. For instance, chidamide is being tested in combination with PD-1 blocking antibodies, lenalidomide, and gemcitabine in phase IV trials treating peripheral T cell lymphoma (ClinicalTrials.gov NCT04040491).

### Lysine demethylase inhibitors (KDMis)

Caffeic acid is an inhibitor of the histone demethylase KDM4C/JMJD2C, also known as gene amplified in squamous cell carcinoma 1 (*GASC1*). Caffeic acid is currently in a phase III trial for advanced esophageal squamous cell cancer (NCT04648917, NCT03070262). Targeting of the histone demethylase LSD1, which is highly expressed in lymphoblasts in acute myeloid leukemia (AML) patients, appears promising. Tranylcypromine (TCP), an irreversible monoamine oxidase inhibitor, also inhibits KDM1A/LSD1, and has been used as an antidepressant and anti-anxiolytic agent for half a century. In a recent phase I/II trial, TCP combined with all-*trans* retinoic acid induced differentiation of blasts in refractory/relapsed AML patients, leading to improved clinical outcomes (147).

## Figures and Tables

**Table 1:**
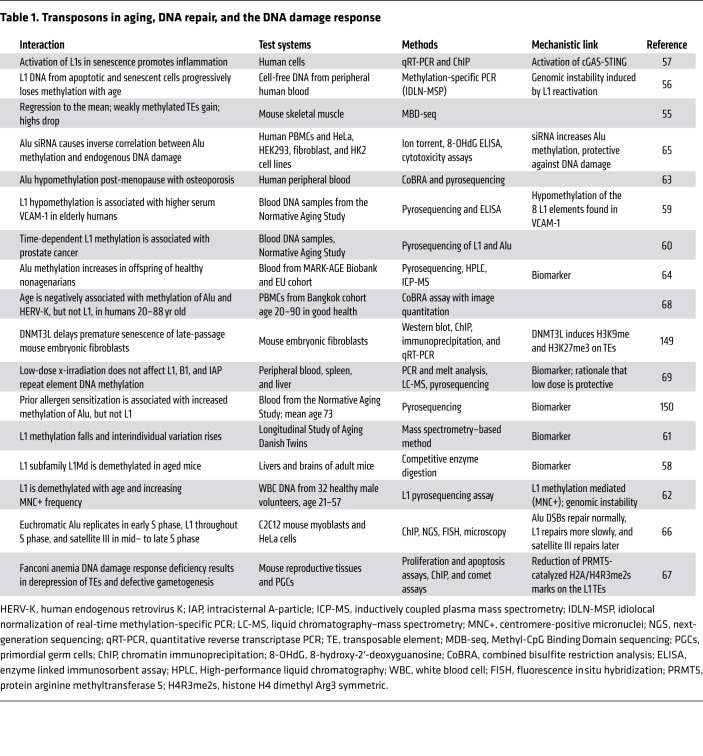
Transposons in aging, DNA repair, and the DNA damage response

**Table 2:**
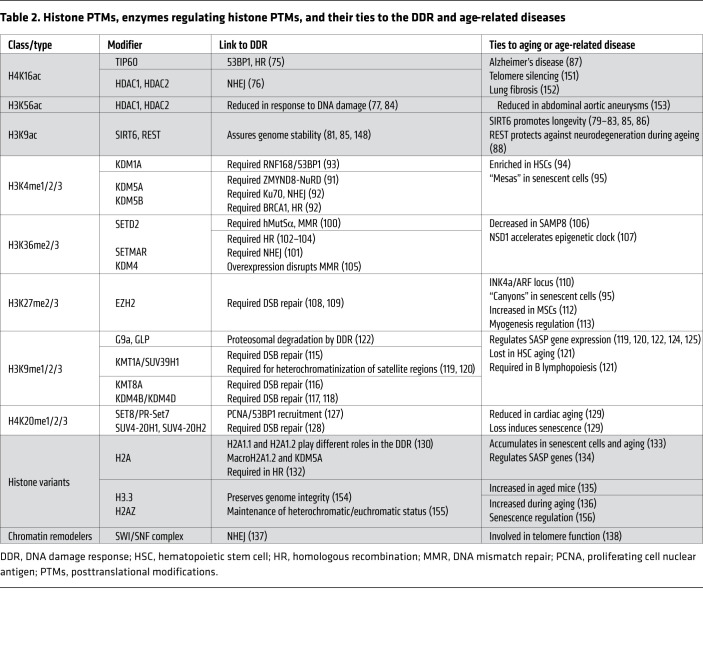
Histone PTMs, enzymes regulating histone PTMs, and their ties to the DDR and age-related diseases

## Version 1 08/15/2022

Electronic publication
